# Critical Assessment of Information Extraction Systems in Biology

**DOI:** 10.1002/cfg.337

**Published:** 2003-12

**Authors:** Christian Blaschke, Lynette Hirschman, Alexander Yeh, Alfonso Valencia

**Affiliations:** 1 Protein Design Group CNB/CSIC Madrid Spain; 2 The MITRE Corporation Bedford MA 01730 USA

## Abstract

An increasing number of groups are now working in the area of text mining, focusing on a wide range of problems and applying both statistical and linguistic approaches. However, it is not possible to compare the different approaches, because there are no common standards or evaluation criteria; in addition, the various groups are addressing different problems, often using private datasets. As a result, it is impossible to determine how well the existing systems perform, and particularly what performance level can be expected in real applications. This is similar to the situation in text processing in the late 1980s, prior to the Message Understanding Conferences (MUCs). With the introduction of a common evaluation and standardized evaluation metrics as part of these conferences, it became possible to compare approaches, to identify those techniques that did or did not work and to make progress. This progress has resulted in a common pipeline of processes and a set of shared tools available to the general research community. The field of biology is ripe for a similar experiment. Inspired by this example, the BioLINK group (Biological Literature, Information and Knowledge [1]) is organizing a CASP-like evaluation for the text data-mining community applied to biology. The two main tasks specifically address two major bottlenecks for text mining in biology: (1) the correct detection of gene and protein names in text; and (2) the extraction of functional information related to proteins based on the GO classification system. For further information and participation details, see http://www.pdg.cnb.uam.es/BioLink/BioCreative.eval.html


**Comparative and Functional Genomics**
*Comp Funct Genom* 2003; **4**: 674–677.
Published online in Wiley InterScience (www.interscience.wiley.com). **DOI:** 10.1002/cfg.337

**Conference Review**

### Critical assessment of information extraction systems in biology

Christian Blaschke[1]*, Lynette Hirschman[2], Alexander Yeh[2] and Alfonso Valencia[1]
[1] *Protein Design Group, CNB/CSIC, Madrid, Spain*
[2] *The MITRE Corporation, Bedford, MA 01730, USA*

**Correspondence to: Christian Blaschke, Protein Design Group, CNB/CSIC, Madrid, Spain. E-mail: blaschke@cnb.uam.es*

Received: 18 September 2003
Revised: 24 September 2003
Accepted: 25 September 2003

### Abstract

**An increasing number of groups are now working in the area of text mining, focusing on a wide range of problems and applying both statistical and linguistic approaches. However, it is not possible to compare the different approaches, because there are no common standards or evaluation criteria; in addition, the various groups are addressing different problems, often using private datasets. As a result, it is impossible to determine how well the existing systems perform, and particularly what performance level can be expected in real applications. This is similar to the situation in text processing in the late 1980s, prior to the Message Understanding Conferences (MUCs). With the introduction of a common evaluation and standardized evaluation metrics as part of these conferences, it became possible to compare approaches, to identify those techniques that did or did not work and to make progress. This progress has resulted in a common pipeline of processes and a set of shared tools available to the general research community. The field of biology is ripe for a similar experiment. Inspired by this example, the BioLINK group (Biological Literature, Information and Knowledge [1]) is organizing a CASP-like evaluation for the text data-mining community applied to biology. The two main tasks specifically address two major bottlenecks for text mining in biology: (1) the correct detection of gene and protein names in text; and (2) the extraction of functional information related to proteins based on the GO classification system. For further information and participation details, see http://www.pdg.cnb.uam.es/BioLink/BioCreative.eval.html** **Copyright © 2003 John Wiley & Sons, Ltd.**

### Introduction

Researchers in natural language processing (NLP) and information extraction (IE) have for many years now used common evaluations to accelerate their research progress, e.g. via the Message Understanding Conferences (MUCs) and the Text Retrieval Conferences (TRECs). This not only results in the formulation of common goals but also makes it possible to compare different systems, providing a degree of transparency to the field.

The field of bioinformatics also has a tradition of competitions, e.g. in protein structure prediction (CASP [2]) or gene predictions in entire genomes (at the 'Genome-based Gene Structure Determination' symposium held on the Wellcome Trust Genome Campus).

As mentioned above, there has been increasing activity in the field of text mining in biology, including sessions at the Pacific Symposium of Biocomputing, as well as workshops [8,11] and sessions on language and biology in computational linguistics [9,12]. However, systematic evaluation of text mining systems in biology has only just begun, e.g. see the recent evaluation for the KDD cup [7] and the genomics track for this year's TREC conference [15]. We have therefore decided to set up an assessment of text mining systems in biology, by defining a common task, common datasets and a clearly defined evaluation.

Copyright © 2003 John Wiley & Sons, Ltd.

BioCreAtIvE, for Critical Assessment of Information Extraction systems in Biology, is being organized by the BioLINK group [1]. Following the tradition of CASP, the emphasis will be more on the comparison of methods and the community assessment of scientific progress, rather than on the purely competitive aspects.

### Definition of the tasks

### Evaluation philosophy

Our aim is to define 'biologically meaningful' tasks — tasks that would be recognized by biologists as a contribution to their work and that constitute a meaningful challenge for current text mining systems. We have selected tasks where 'gold standard' data for training and test can be made available in sufficiently large quantities with modest investment. This has led us to focus on the use of existing expert-curated data from existing biological databases as a source for gold-standard data.

Through discussion at various meetings over the past several years, we have identified two classes of tasks of interest to both researchers and practitioners. The first of these is 'entity identification' in text — the ability to find mentions of relevant biological entities (genes, proteins, small molecules, chemicals, tissues, etc.) in running text. This task enables accurate indexing of entities within articles; it also takes the first step towards the more ambitious task of relation extraction and, eventually, pathway discovery from the literature. The second task is more ambitious and focuses on the automatic functional annotation of proteins using the Gene Ontology (GO) classes [4].

### Task 1: entity extraction

The goal of the entity extraction task is to assess the ability of an automated system to identify the genes (or proteins, where there is ambiguity) mentioned in text.

The 'natural language processing' or MUC version of this task required that a system identify *each mention* of a gene or protein in the text. The MUC 'named entity' task requires that the system identify all mentions of genes (or proteins or ...) in a text; this is generally done as in-line mark-up of the occurrences of these names, as in the GENIA annotated corpus [5]. It is labour-intensive to provide consistent annotation for this task, because of questions about how much of the name to include (e.g. 'feline homologue of CD2'), what to do with abbreviations embedded in a compound name [e.g. 'VE growth factor (VEGF) receptor-2 (VEGFR-2)'], and what to do with names that may be compound names or conjoined names (e.g. MEK-1/-2).

This aspect (sub-task 1.1) will be evaluated using annotations of gene mentions in sentences from MEDLINE abstracts, provided by Lorraine Tanabe and John Wilbur (NCBI). A second biologically-motivated sub-task (sub-task 1.2) will measure the ability of a system to identify *the list/set of genes* (using unique/standardized gene names) mentioned in passages of text (specifically MEDLINE abstracts curated in model organism databases); this sub-task can be related to sub-task 1.1 by mapping each gene mention to its unique name or symbol. The *gene list* task has the advantage that it is performed by expert human curators in many databases. This means that it is easy to obtain 'ground truth' data for training, by downloading sets of curated articles and the corresponding gene list for each article. For subtask 1.2, we will make available a (reasonably) comprehensive lexicon of standard gene names and their synonyms for the associated model organism. There will be data and resources for identifying gene lists in text from three model organisms: fly, yeast and mouse [3,13,10].

### Task 2: functional annotation of gene products

The second task will address the assignment of GO annotations to human proteins [6]. This is currently done by curators at Swiss-Prot [14] for the human genome, who have agreed to make these annotations available for use as training and test sets.

For this task, the full text of the journal articles will be used, because most of the information is contained in the paper body and not in the abstract alone. The number of publications provided in the training set may be small (most likely a couple of hundred) because full-text will only be provided by a limited number of journals.

The sub-parts of task 2 will be:

1. Selection of relevant papers: detect which papers are relevant for a protein in the sense

Copyright © 2003 John Wiley & Sons, Ltd. 

that they contain information that would be suitable to derive a GO annotation, and provide the evidence text.
2. Provide GO annotation for human proteins: automatically annotate a protein in terms of GO according to the information found in a publication. In addition, find a statement in the text that 'justifies' this annotation.
3. 'Recover' text that supports the GO annotation: find a statement in the text that 'justifies' the database annotation.

The evaluation will mainly be based on the evidence text that is provided. For the second subpart, we will evaluate how close the prediction is in the GO hierarchy to the correct annotation; in this part participants will have to do well in both providing the GO code and identifying correct supporting evidence. Providing only the GO prediction would not be valid; similarly, only providing text but no (or completely incorrect) prediction of the GO code would not be valid.

For all sub-parts, the curators from Swiss-Prot will evaluate how 'useful' the extracted text is for deriving the correct annotation. This usefulness measure is somewhat subjective, but we think that this is realistic, because we want to know how useful a system is under these settings, rather than only how good it is at reproducing a certain way of annotating the training data. We chose this particular set of tasks in consultation with the Swiss-Prot curators, because they are tools that would be of immediate use to them. Since these tasks fit into the current curation pipeline, we plan to quantify the time/cost savings obtainable through interactive semi-automated curation, as systems emerge that can perform these tasks.

### Schedule

- July 2003: Release initial training dataset and initial task guidelines.
- September 2003: Release full training set and revised task guidelines.
- November 2003: Release test data and receive results.
- December 2003: Tabulate results.
- April 2004: Convene final workshop in Granada, Spain.

### Issues to take into account

#### Text source: abstracts vs. full articles

The choice of full paper vs. abstract affects cost, data quantity and data quality. Abstracts are readily available (in large quantities) via PubMed, in a standard ASCII format. On the other hand, most (although not all) curation is done on full text, not just on the abstract. However, full text is more difficult to obtain, because of the absence of a central repository like PubMed, and because of copyright issues. In addition, there are problems with differences in typography, as well as variable document structures and formats (PDF, HTML, XML, etc.). Thus, abstracts are much easier to work with, but full text is probably more realistic.

### Different databases — different focus

Each database has its specific focus, e.g. for gene products, FlyBase [3] only curates information about wild-type alleles of genes. For genes and alleles, information on mutants is also curated. In general, genes from other organisms, or genes mentioned as background, do not appear in the gene lists and are not curated. This raises the possibility that an automated system might correctly detect a gene mentioned in an abstract, but that this gene might not appear in the gene list generated by the curators.

### The gene nomenclature is constantly changing

Genes are constantly being discovered and named. To assist developers, we have taken a snapshot of the nomenclature resources of the three databases that we are using and have processed each one to assemble a lexicon: a comprehensive list of gene names and synonyms for the particular organism. This is intended to make things easier for developers. However, because it is only a snapshot, it is almost immediately out of date. This means that there may also be novel names that are not in the lexicon and have no canonical form.

### Final thoughts

BioCreative is, we believe, the first biologically motivated evaluation of text mining systems. However, we are aware that only history can judge the

Copyright © 2003 John Wiley & Sons, Ltd.

impact of our efforts and this may be the first, but almost certainly not the last, initiative of this type.

There are many relevant problems that we are not addressing at this time, e.g. detection of other entities, such as chemical substances or tissue and cell types; detection of relations among entities, such as protein–protein, gene–disease or disease–symptom relations; and tasks going beyond fact extraction, such as classification and summarization of information for a given protein. We expect that if there are continued evaluations, they will be extended to cover these critical areas.

Until very recently, text mining in biology has been performed primarily on abstracts. We hope to demonstrate that crucial information is contained in the body of the publications and that the access to full text will be crucial for further development of text mining systems.

### References

1. Biological literature, information and knowledge: **http://www.pdg.cnb.uam.es/BioLINK/**.
2. Critical assessment of techniques for protein structure prediction: **http://predictioncenter.llnl.gov/**.
3. Flybase: **http://flybase.bio.indiana.edu/**.
4. Gene Ontology Consortium: **http://www.geneontology.org/**.
5. GENIA: **http://www-tsujii.is.s.u-tokyo.ac.jp/GENIA/**.
6. GO annotation for human: **http://www.ebi.ac.uk/GOA/release.html**.
7. Knowledge Discovery and Data Mining Cup 2002. **http://www.biostat.wisc.edu/~craven/kddcup/**.
8. Language modeling of biological data, University of Pennsylvania, February 2001. **http://www.ircs.upenn.edu/modeling2001/**.
9. Language processing and biological data special session, Human Language Technology Workshop, March 2002.
10. Mouse genome informatics, the Jackson Laboratory: **http://www.informatics.jax.org**.
11. Natural language processing and ontology building workshop, University of Tokyo, February 2002. **http://www-tsujii.is.s.u-tokyo.ac.jp/GENIA/WS.html**.
12. Natural language processing in the biomedical domain workshops, Association of Computational Linguistics, July 2002 and July 2003: **http://www.ccs.neu.edu/home/futrelle/bionlp/acl02/BIO/**; **http://www-tsujii.is.s.u-tokyo.ac.jp/ACL03/bionlp.htm**.
13. Saccharomyces Genome Database: **http://www.yeastgenome.org/**.
14. Swiss-Prot: **http://www.ebi.ac.uk/swissprot/**.
15. Text Retrieval Conference (TREC): **http://trec.nist.gov/**.

Copyright © 2003 John Wiley & Sons, Ltd. 

